# Constructing a folate metabolism gene signature for predicting prognosis in pulmonary neuroendocrine carcinomas

**DOI:** 10.7150/jca.102186

**Published:** 2024-10-14

**Authors:** Quanying Tang, Luoyi Li, Ruiyao Wang, Xin Jin, Xuewang Jia, Yifan Zhu, Xiaoyue Sun, Jianguo Zhong, Huangsheng Xie, Yurong Da, Lingling Zu, Song Xu

**Affiliations:** 1 Tianjin Key Laboratory of Lung Cancer Metastasis and Tumor Microenvironment, Lung Cancer Institute, Tianjin Medical University General Hospital, Tianjin 300052, China.; 2 Department of Lung Cancer Surgery, Tianjin Medical University General Hospital, Tianjin 300052, China.; 3 Tianjin Institute of Immunology, Key Laboratory of Immune Microenvironment and Disease of the Ministry of Education, The Province and Ministry Co-sponsored Collaborative Innovation Center for Medical Epigenetics, State Key Laboratory of Experimental Hematology, Department of Immunology, Tianjin Medical University, Tianjin 300070, China.; 4 Department of Thoracic Surgery, Affiliated Hospital of Hebei University, Baoding 071000, China.

**Keywords:** Pulmonary neuroendocrine tumors, One carbon metabolism, immune microenvironment

## Abstract

Folate metabolism is a crucial biological process in cell proliferation and exhibits its pro-tumorigenic functions in multiple tumor types. However, its role in pulmonary neuroendocrine carcinomas remains uncertain. Folate metabolism related genes were obtained from previous studies, and the gene expression data and clinical data were collected from GEO database. The expression patterns of folate metabolism related genes were measured across normal and tumor tissues. We subsequently assessed their prognostic role using Kaplan-Meier and univariate Cox regression analysis. The core genes were isolated from 16 prognostic genes through four algorithms. Based on the expression of core genes, patients were divided into two clusters employing consensus clustering algorithm. Furthermore, we evaluated immune infltration level, biological mechanisms, and drug sensitivity. ALDH1L2 was finally identified through qRT-PCR and its pro-tumorigenic function was confirmed via *in vitro* experiments. The expression patterns of 26 folate metabolism related genes were evaluated between normal lung tissues and PNEC tumor tissues, and 20 of them exhibited differential expression. All of folate metabolism related genes were related to the prognosis of PNECS and 16 genes were identified as prognostic genes. Using SVM-RFE, RF, Xgboost and LASSO algorithm, three core genes were isolated from 16 prognostic genes. Based on the expression patterns of core genes, PNECs patients were divided into two clusters through consensus clustering algorithm. Cluster 1 was characterized by the worse survival, higher immune infiltration level, and sensitivity to chemotherapy. Compared with the HBEC cells, ALDH1L2 was notably overexpressed in NCI-H446 cells (SCLC cell line). ALDH1L2 knockdown significantly repressed the proliferation and migration capacity of tumor cells and increased the cell proportion in S phase. Our results indicated that folate metabolism gene signature is a reliable biomarker for PNECs. Classification based on this signature could be utilized to guide the treatment of PNECs patients and improve its prognosis.

## Introduction

Pulmonary neuroendocrine carcinomas (PNECs) are a type of tumor derived from pulmonary endocrine cells, roughly accounting for 25% of all lung tumors^1^. PNECs comprise four heterogeneous neoplasms, including small cell lung cancer (SCLC), large cell neuroendocrine carcinoma (LCNEC), and two pulmonary carcinoids (PCs), antypical carcinoid (AC) as well as typical carcinoid (TC)^2^. Among these, SCLC is the most deleterious subtype and is characterized by the worst survival, followed by LCNEC, AC, and TC^3^. While surgical resection serves as the primary treatment for early-stage PNECs, effective therapies for advanced tumors notably continue to be deficient^4^, ^5^.

Folate metabolism, also referred to as one-carbon (1C) metabolism, plays a crucial role in cell proliferation^6^. Folate metabolism provides essential 1C units to facilitate various biological processes, including biosynthesis of nucleotide, amino acid homeostasis, and epigenetic regulation^7^, ^8^. Disruptions in folate metabolism can cause several diseases, such as neural tube defects, anemia, and cancer. The abnormal expression of folate metabolism-related enzymes is closely related to the occurrence and development of various cancers and has a prognostic role^9^-^11^. The therapeutic benefits derived from using the folate antagonist aminopterin, which successfully impeded tumor cell proliferation in patients with acute lymphoblastic leukemia, ratified the significance of folate metabolism in cancer treatment^12^. Since then, several folate metabolism inhibitors that target folate metabolism were developed, such as 5-fluorouracil (5-FU), methotrexate and pemetrexed^8^. 5-FU is a inhibitor of thymidylate synthase (TYMS), a key enzyme in folate metabolism, and directly suppress the proliferation of tumor cells^13^. The anti-tumor role of methotrexate is exerted most potently by blocking dihydrofolate reductase (DHFR), while pemetrexed has a multi-targeting role, acting on three key folate metabolism enzymes: TYMS, DHFR, and glycinamide ribonucleotide formyltransferase (GART)^8^, ^14^, ^15^. In addition, previous studies has recognized that other folate metabolism enzymes could promote the occurrence and development of multiple tumor types. For instance, MTHFD2, which shows high expression within hepatocellular carcinoma and colorectal cancer, is associated with a shorter survival time^16^, ^17^. Likewise, the expression level of SHMT2 is elevated in colorectal and lung cancer tissues, and the overexpressed SHMT2 promote the tumor progression^18^, ^19^.

Apart from malignant cells, folate metabolism also play a pivotal role in the development and differentiation of immune cells, which involves the activation of T cells and macrophages^20^, ^21^. In recent years, it has been established that the tumor immune microenvironment (TIME), significantly impacts tumor development, prognosis, and treatment^22^. T cells and macrophages constitute vital components of TIME. They are not only intricately linked to the proliferation, differentiation, and demise of tumor cells, but also play a significant role in tumor chemotherapy, radiotherapy, and immunotherapy^23^-^26^. Moreover, the safety and effectiveness of combined immunotherapy and chemotherapy in small cell lung cancer (SCLC) were validated in a clinical trial, showing an extended median overall survival (OS)^27^. Nevertheless, the standard therapy for large cell neuroendocrine carcinoma (LCNEC) and pulmonary carcinoids remains inadequate, while the effectiveness of immunotherapy and chemotherapy remains uncertain.

**Figure 1:**
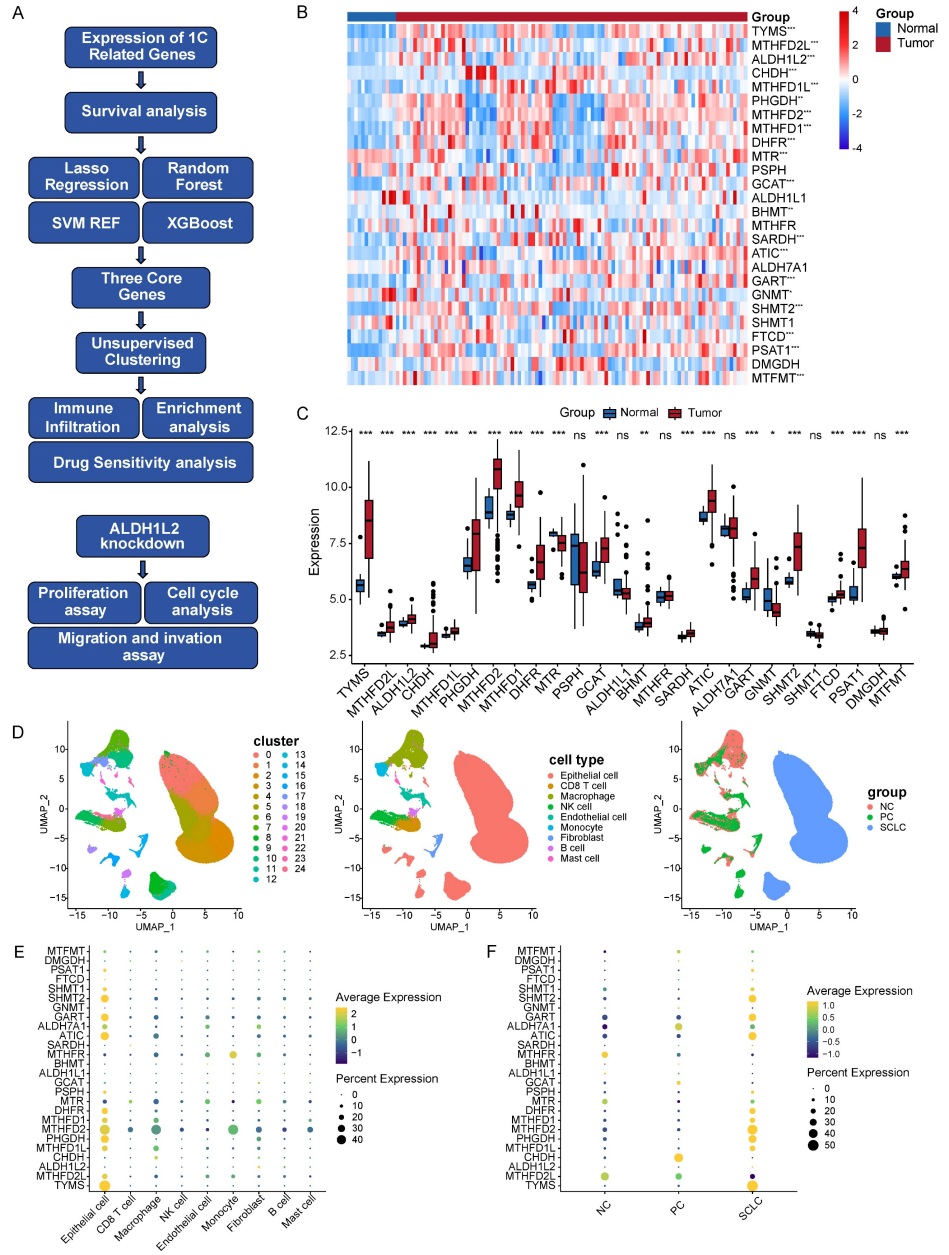
Program flowchart and expression patterns of folate metabolism related genes. A Program flowchart for this study. B The heatmap shows the expression patterns of 26 folate metabolism related genes. C The box plot shows the expression patterns of 26 folate metabolism related genes. D The clusters, cell types, and sample type annotation of 244,076 cells using UMAP plots. E Dot plots of folate metabolism related genes across cell types. F Dot plots of folate metabolism related genes in epithelial cells across sample types. *P<0.05, **P<0.01, ***P<0.001.

In our current study, we utilize transcriptome and single-cell RNA-sequencing (scRNA-seq) data to investigate the role and underlying mechanism of folate metabolism in PNECs (Figure 1A). First, we evaluated the expression levels of crucial enzymes in folate metabolism using transcriptome and scRNA-seq data. Second, we assessed the association of these key enzymes with the prognosis of LCNECs. Third, we utilized four machine-learning algorithms to select the core genes, from which we constructed a gene signature. Subsequently, we evaluated the immune cell infiltration level, drug sensitivity, and biological mechanism across two sub-types. Finally, we found that ALDH1L2 is highly expressed in SCLC cell lines and promoted its malignant phenotype.

## Materials and methods

### Data collection and processing

Only one transcriptome dataset (GSE30219) and two scRNA-seq datasets (GSE216182, GSE196303) of PNEC were download from GEO database (https://www.ncbi.nlm.nih.gov/geo/). Patients with pathological types of small cell lung cancer, lung large cell neuroendocrine carcinoma, and lung carcinoid were selected from GSE30219 for subsequent research. The bulk RNA-seq data was normalized through “limma” package and 115 samples was finally collected according to clinical information, including normal tissue (n = 14), SCLC (n = 21), LCNECs (n = 56), and PCs (n = 24). The scRNA-seq datas were converted to three seurat objects by utilizing “Seurat” package, including normal lung tissue (n = 10), SCLC (n = 16), PCs (n = 3). Firstly, cells were excluded with different threshold values described in below: (1) gene expression exceeding 4000 or below 200; (2) percentage of mitochondrial gene expression exceeding 20%, 30%, and 50% for normal lung tissue, SCLC, and PCs, respectively. Subsequently, all samples were merged using “Seurat” package, and the batch effect was removed by “harmony” package. Thirdly, top 5000 variable genes were identified for data normalization using the FindVariableFeatures function in the Seurat package. All genes were included to scale the expression data, and then principal component analysis (PCA) was performed based on top 5000 variable genes. Next, uniform manifold approximation and projection (UMAP) algorithms were employed for dimensionality reduction and visualization. Finally, major cell types were identified according to the expression of specific markers. The folate metabolism related genes were similarly determined in our previous study^28^.

### Prognostic analysis and molecular classification

The prognostic value of 26 folate metabolism related genes was assessed through Kaplan-Meier analysis based on the optimal cutoff point, and two R packages (“survival” and “survminer”) were utilized in this algorithm. In addition, univariate Cox proportional hazard regression analysis was also employed to evaluate the prognostic value of them. Genes with p < 0.05 in the Kaplan-Meier analysis and univariate Cox proportional hazard regression analysis were considered prognosis-related genes. Four machine learning algorithms were used to identified the core genes from prognosis-related genes, including support vector machine recursive feature election (SVM-RFE), random forest (RF), Extreme Gradient Boosting (Xgboost), and Least absolute shrinkage and selection operator (LASSO). The results of each four machine learning methods were intersected and the core gene set was generated (*ALDH1L2, MTHFD2, SHMT2*). The consensus clustering algorithm was utilized to identify the sub-types in PNECs samples based on the expression of core genes and the R package “ConsensusCluster” was employed to support this process. The parameters of consensus clustering were set up as described below: the number of repetitions = 1,000 bootstraps; resample rate = 0.8. The 101 PNECs samples were finally gathered into two sub-types, cluster 1 (n = 78) and cluster 2 (n = 23).

### Identifcation of DEGs and WGCNA

To identify the diferentially expressed genes (DEGs) between two clusters, the R package “limma” was applied to calculate the differential expression of genes. |Log(2) fold change|>0.5 and adjusted p<0.05 were set as the criteria for DEGs (n = 6332). According to the DEGs expression profile, a weighted gene co-expression network analysis (WGCNA) was constructed by utilizing the R package "WGCNA". The soft threshold was set to 5 and the weighted adjacency matrix was transformed into a topological overlap matrix (TOM) to calculated the network connectivity. Next, the hierarchical clustering algorithm was employed to build the cluster tree structure of the TOM matrix. Both of different branches of the cluster tree and different colors represent the different gene modules. All the DEGs were finally gathered into multiple modules, and the association between the gene modules and phenotype was assessed.

### GO, KEGG, and GSEA analyses

For further exploring the underlying biological mechanisms between two clusters, three gene enrichment algorithms were performed to analyze the genes from two modules. R “clusterProfler” and “org.Hs.eg.db” were employed in the process of Gene Ontology (GO) and Kyoto Encyclopedia of Genes and Genomes (KEGG) analyses. In addition, gene set enrichment analyses (GSEA) were also conducted based on the Hallmark gene set “c5.all.v7.0.entrez.gmt” of MSigDB by using the same R package.

### Analysis of tumor immune microenvironment

We firstly evaluated the infiltration level of immune cells through four methods, and then the expression level of 50 immune check points were calculated. CIBERSORT (http://cibersort.stanford.edu/) was utilized to calculated the abundances of 22 immune cell types, and the infiltration level of 64 immune and stromal cell types was obtained from xCell, a webtool (http://xcell.ucsf.edu/) which provides cell type enrichment analysis service. A single-sample gene set enrichment analysis (ssGSEA) provided the score of 28 immune cell types, and the MCP-counter algorithm was also employed to the abundances of 10 immune cell types. R packages “GSVA” and “IOBR” were utilized for the two algorithms respectively. Next, we assessed the expression level of 25 HLA family members, and estimated the immune score through a R package “estimate”.

### Drug sensitivity analysis

The expression data of 805 cell lines and IC50 data of 198 drugs were downloaded from The Genomics of Drug Sensitivity in Cancer (GDSC) database (https://www.cancerrxgene.org/), and the information of 829 cell lines and 545 drugs was obtained from The Cancer Therapeutics Response Portal (https://portals.broadinstitute.org/). We utilized the R package “oncoPredict” to predict the drug sensitivity of each LNECs sample. Then, we compared the differences between two sub-types through the R package “limma”, and the drugs with the p<0.05 were selected.

### Cell culture and transfection

Small cell lung cancer cells (H446, H526, H69), pulmonary carcinoid cells(H720, H727), and normal ovary cells (HBEC) were obtained from the American Type Culture Collection (ATCC, Manassas, VA, USA). These cells were cultured in RPMI 1640 medium (Gbico, Thermo Fisher Scientific) with 10% fetal bovine serum (FBS) (Nemzerum, New Zealand), 100 IU/mL penicillin, and 10 µg/mL streptomycin (Gbico, Thermo Fisher Scientific). All cells were cultured at 37°C with 5% CO2. The small interfering RNA (siRNA) was employed to knock down the gene expression and the ALDH1L2 siRNA was obtained from Shanghai IBSBIO (IBSBIO, Shanghai, China). The specifc sequence was in below: siALDH1L2: 5'-GAGAUCAUGUGAUGUUGAACC-3'. The siALDH1L2 was transfected into the NCI-H446 cell line with Lipofectamine™ 2000 (Invitrogen) according to the manufacturer's protocol.

### Quantitative real time-PCR (qRT-PCR)

After 48h-72h from cell transfection, total RNA of SCLC and PCs cell lines were extracted with Trizol reagent (Termo Fisher, 16096020, USA). PrimeScriptTM RT Master Mix (Takara) was utilized to reversely transcribe the extracted RNA. The qRT-PCR process was performed with AceQ qPCR SYBR Green Master Mix (Vazyme, Nanjing, China). The Ct values were then obtained, and the relative mRNA expression of target genes was calculated employing the 2^-∆∆Ct^ method. Primer sequences are listed in below:

ALDH1L2 forward primer: 5′-GCTGAAGTTGGCACTAATTGGC′, reverse primer: 5′-TGAACACCCCTACTACTCGGT′.

MTHFD2 forward primer: 5′-GATCCTGGTTGGCGAGAATCC-3′, reverse primer: 5′-TCTGGAAGAGGCAACTGAACA-3′.

SHMT2 forward primer: 5′-CCCTTCTGCAACCTCACGAC-3′, reverse primer: 5′-TGAGCTTATAGGGCATAGACTCG-3′.

### Proliferation analysis

For the Cell Counting Kit-8 (CCK-8) assay, after 48h-72h transfection, cells were seeded into 96-well plates with a density of 3000 cells/well and maintained for 24, 48, 72, and 96 hours at 37 °C with 5% CO2. Then, 20 μl CCK-8 (K1018-5ml, APExBIO, Shanghai, China) was added to each well for 4 hours at 37 °C with 5% CO2. The absorbance value was then measured at 450 nm every 24 h, and the experiment lasted for 96 h. Each sample was performed in triplicate.

Ethynyl2'-deoxyuridine (EdU) assay was also performed to assess the proliferation ability. After 48h-72h transfection, cells were seeded into 96-well plates and maintained at 37 °C with 5% CO2. 10 μM EdU (Beyotime, BeyoClick™EDU-555, China) was added into per well for 2 h, and then the Apollo and Hoechst solutions were used to fix and stain the cells.

### Cell cycle analysis

For cell cycle assays, after 48h-72h transfection, treated cells are collected, rinsed twice with cold PBS, and then fixed in 70-90% ethanol overnight at 4 degrees Celsius. After fixation, the cells were washed with PBS, followed by resuspension with 500 μL of propidium iodide (PI) solution (BD, Biosciences, USA) and incubated for 30 min away from light. Finally, the cell cycle was measured by flow cytometry.

### Transwell migration and invasion assay

After 48-72 hours, transfected cells were resuspended in serum-free medium for preparing cell suspension. 24-well Transwell chambers (BD Biosciences, San Diego, CA, USA) were pre-treated with or without Matrigel (Corning, NY, USA). 5×10^4^ cells resuspended with 400 ml of serum-free medium were added to the upper chamber, and after that 500 µl of medium containing 10% fetal bovine serum was added to the lower chamber. Matrigel is added to the upper chamber for invasion experiments, but not for migration experiments. After 48h/24h of incubation, the invaded /migrated cells were fixed with 4% paraformaldehyde, stained with 1% crystal violet, and photographed under a microscope.

### Statistical analysis

Statistical tests were performed using R software (version 4.2.2), SPSS 22.0 (IBM, NY, United States), and GraphPad Prism 9.0. For quantitative data, Student's t-test or Wilcoxon rank-sum test were utilized to compare the differences between two groups. Categorical variables were analyzed using two-sided Fisher's exact tests. All statistical tests were bilateral, and P <0.05 was considered statistically significant.

## Results

### Expression of folate metabolism related genes in PNECs

This study examined the expression patterns of 26 genes related to folate metabolism in PNECs tumor tissues and normal lung tissues, utilizing bulk RNA-seq data. Out of the 26 genes, differential expression was observed in 20 genes when comparing tumor to normal tissues (Figure 1B and 1C). More specifically, *PHGDH*,* PSAT1*,* FTCD*,* SHMT2*,* MTHFD2L*,* MTHFD2*,* MTHFD1L*,* MTHFD1*,* GCAT*,* SARDH*,* BHMT*,* CHDH*,* TYMS*,* GART*,* ATIC*,* ALDH1L2*,* DHFR*, and* MTFMT* showed elevated expression in tumor tissues. Conversely, *MTR* and *GNMT* were found to be expressed at significantly lower levels in tumors. To gain a more in-depth understanding of these expression patterns at a cellular level, we analyzed scRNA-seq data sourced from PNECs, selecting 244,076 cells derived from 29 samples. These cells consequently underwent classification into 25 distinct clusters, which corresponded to 9 major cell types, including epithelial cells, CD8 T cells, macrophages, and endothelial cells (Figure 1D). The epithelial cells of SCLC were biologically differentiated from epithelial cells found in PCs and normal lung tissue (Figure 1D). Subsequently, the expression pattern of the folate metabolism associated genes was examined, revealing elevated expression mainly in epithelial cells, as opposed to immune or stromal cells (Figure 1E). The expression pattern of folate metabolism related genes in PCs epithelial cells significantly diverged from that in SCLC, but more resembled the normal lung tissue (Figure 1F).

### Prognostic value of folate metabolism related genes in PNECs

**Figure 2:**
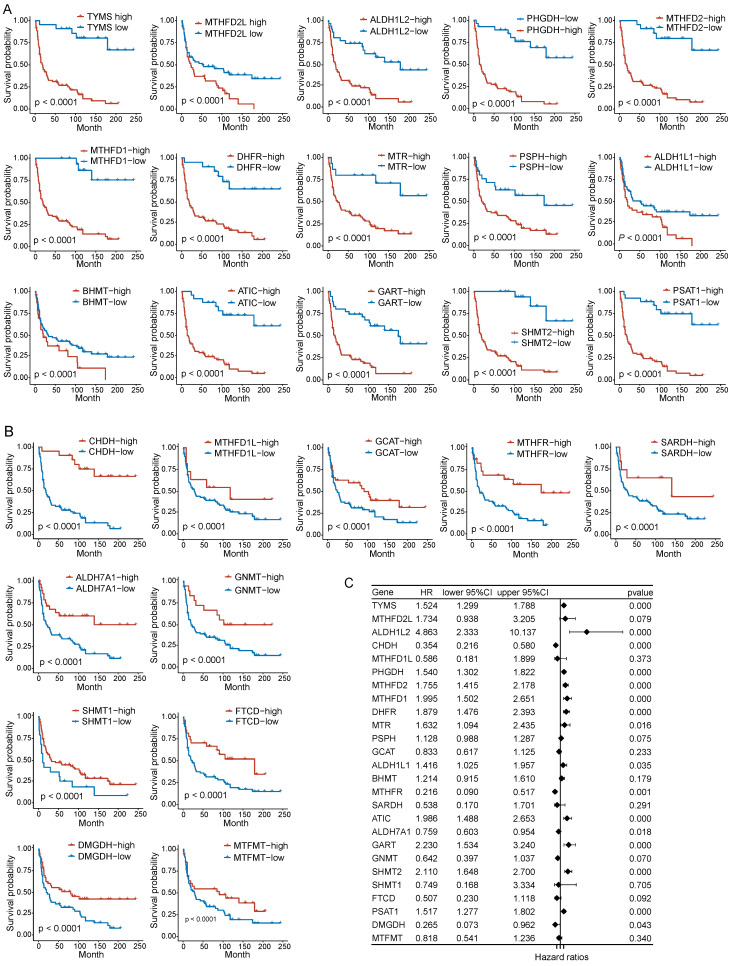
Prognostic analysis in OS of folate metabolism related genes. A Kaplan-Meier curves show 15 genes are associated with a shorter OS. B Kaplan-Meier curves show 11 genes are related to a longer OS. C Univariate Cox regression analysis revealed 16 genes were associated with OS.

**Figure 3:**
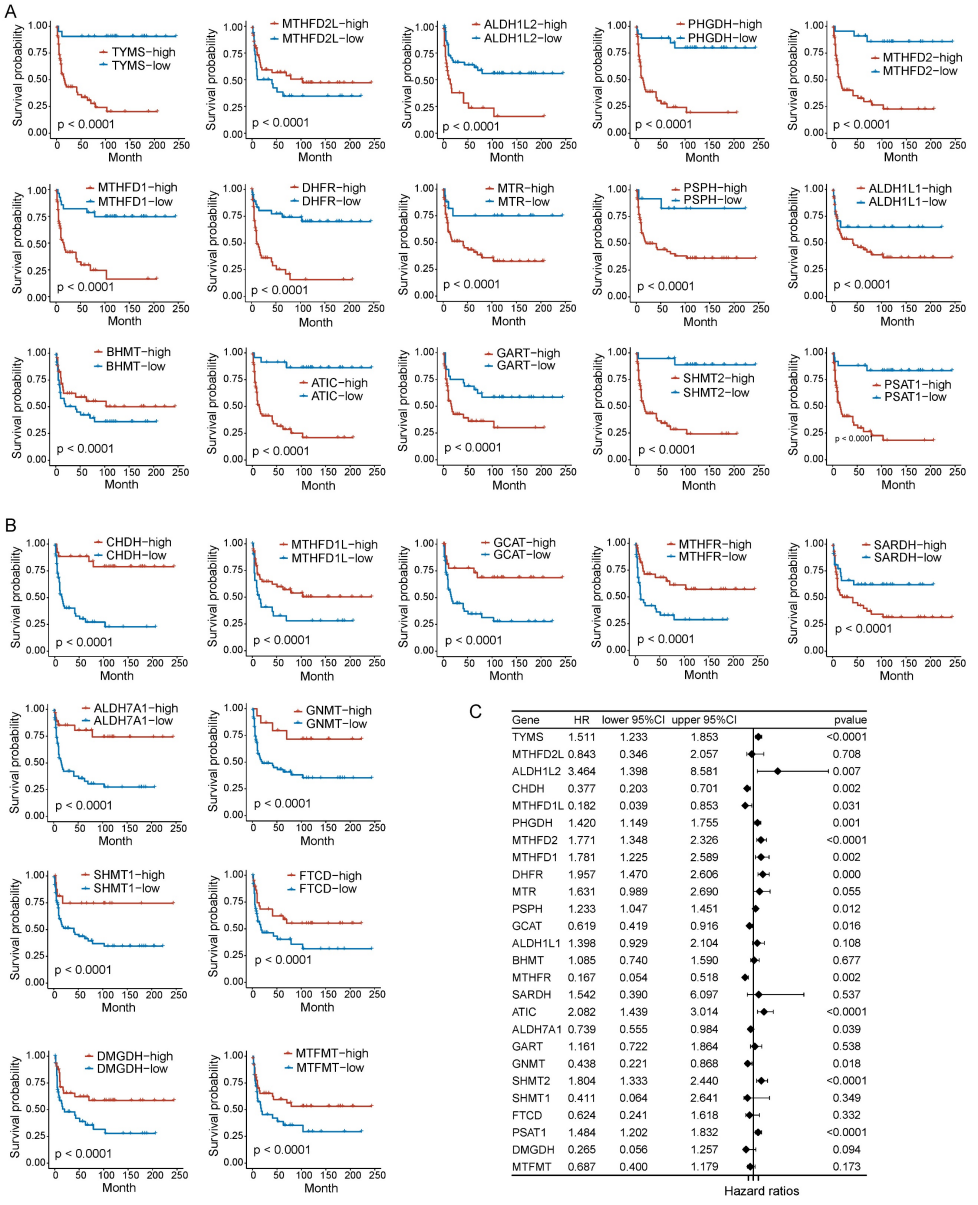
Prognostic analysis in PFS of folate metabolism related genes. A PFS Kaplan-Meier curves of 15 shorter OS related genes. B PFS Kaplan-Meier curves of 11 longer OS related genes. C Univariate Cox regression analysis revealed 16 genes were associated with PFS.

To assess the correlation between folate metabolism related genes and PNECs prognosis, both Kaplan-Meier analysis (based on optimal cutoff) and univariate Cox regression analysis were utilized. The Kaplan-Meier analysis demonstrated an association between all examined folate metabolism related genes and the overall survival (OS) of PNECs. More specifically, 15 of these genes were associated with a shorter OS, whilst 11 were correlated with positive outcomes (Figure 2A and 2B). The findings of the univariate Cox regression analysis further substantiated this result, with nine genes being excluded in this process (Figure 2C). Correspondingly, the 26 genes related to folate metabolism were also correlated with progression-free survival (PFS). Amongst these, 14 were identified as potential risk factors for PFS, with 12 acting as protective factors (Figure 3A and 3B). The Cox regression analysis proposed that 16 genes could significantly influence the PFS of PNECs (Figure 3C). Interestingly, contradictory prognostic values were observed for three genes, *MTHFD2L*, *BHMT*, and *SARDH*, between OS and PFS.

### Identification of core genes and molecular classification

**Figure 4:**
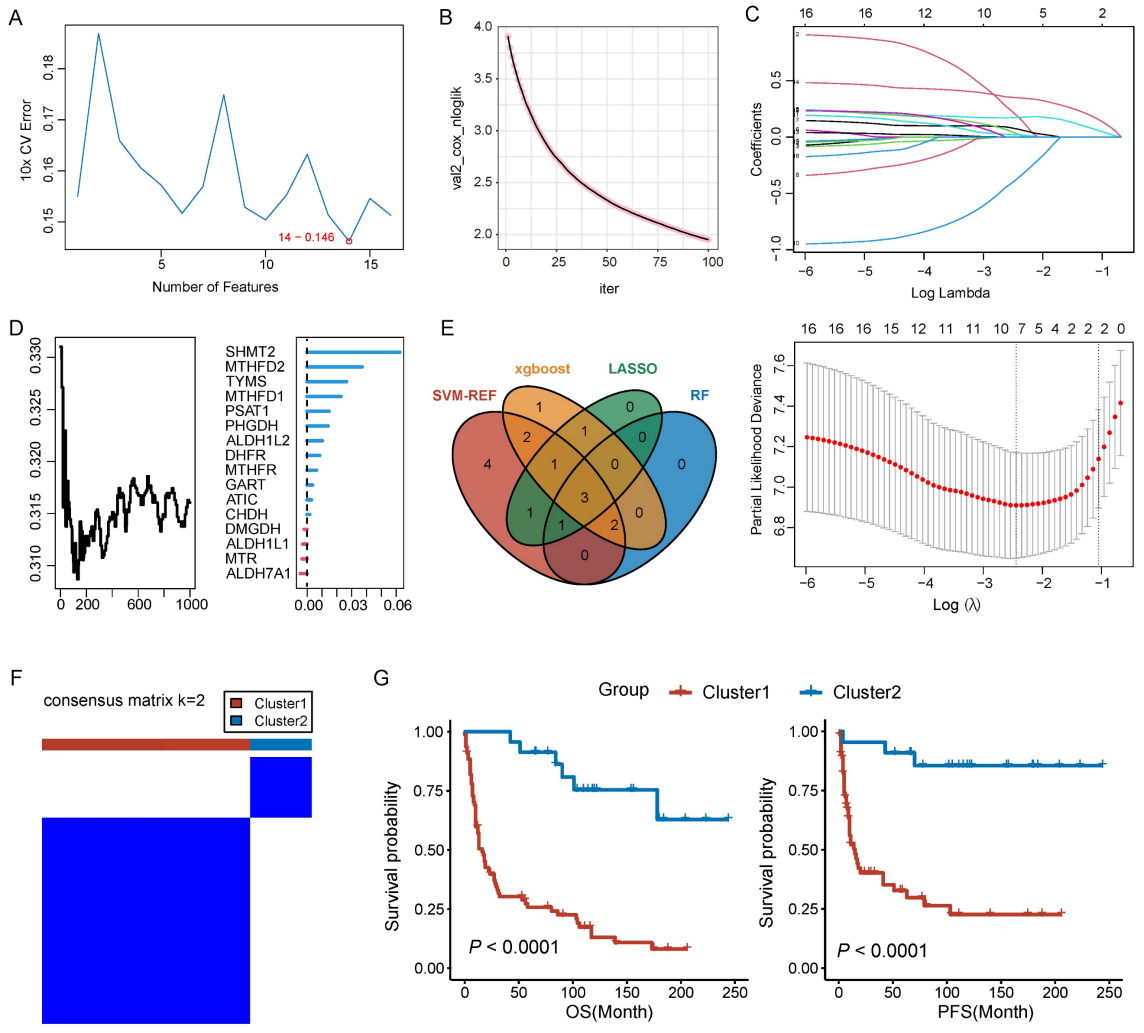
Identifcation of core genes and construction of molecular classification. A Prognostic genes were screened based on the SVM-RFE algorithm. B Prognostic genes were filtered by XGBoost algorithm. C Selection of prognostic genes using LASSO algorithm. D Screening of prognostic genes through RF algorithm. E Venn plot for the core genes isolated by SVM-RFE, and XGBoost algorithms, LASSO regression and RF. F Consensus matrix plots for k=2. G The Kaplan-Meier curves of OS and PFS across the two clusters.

In pursuit of identifying core genes of folate metabolism in PNECs, four distinct algorithms were used to evaluate the importance of 16 prognosis-related genes. According to the SVM-RFE, RF, and LASSO results, 14, 6, and 7 genes respectively were selected by these methods (Figure 4A, 4B and 4C). Additionally, the top ten genes derived from the Xgboost algorithm were also included for further consideration (Figure 4D). Subsequently, by integrating the genes singled out by each method, three core genes were identified: *MTHFD2*, *SHMT2*, and *ALDH1L2* (Figure 4E). The expression patterns of these core genes in scRNA-seq data were explored (Supplement Figure 1A). Based on the expression patterns of these core genes, a consensus clustering algorithm was employed to construct a molecular classification of PNECs, subdividing all PNECs patients into two distinct sub-types, designated cluster 1 and cluster 2 (Figure 4F). It was observed that patients within cluster 1 exhibited considerably longer overall survival (OS) and progression-free survival (PFS) than those within cluster 2 (Figure 4G). The expression patterns of folate metabolism were different across two clusters (Supplement Figure 1B). The clinical features of the two clusters are presented in the subsequent table. Notably, cluster 1 was characterized by a higher proportion of male patients, an advanced T stage, and a larger number of SCLC and LCNEC samples (Table 1).

### Immune infiltration features of LNECs sub-types

**Figure 5:**
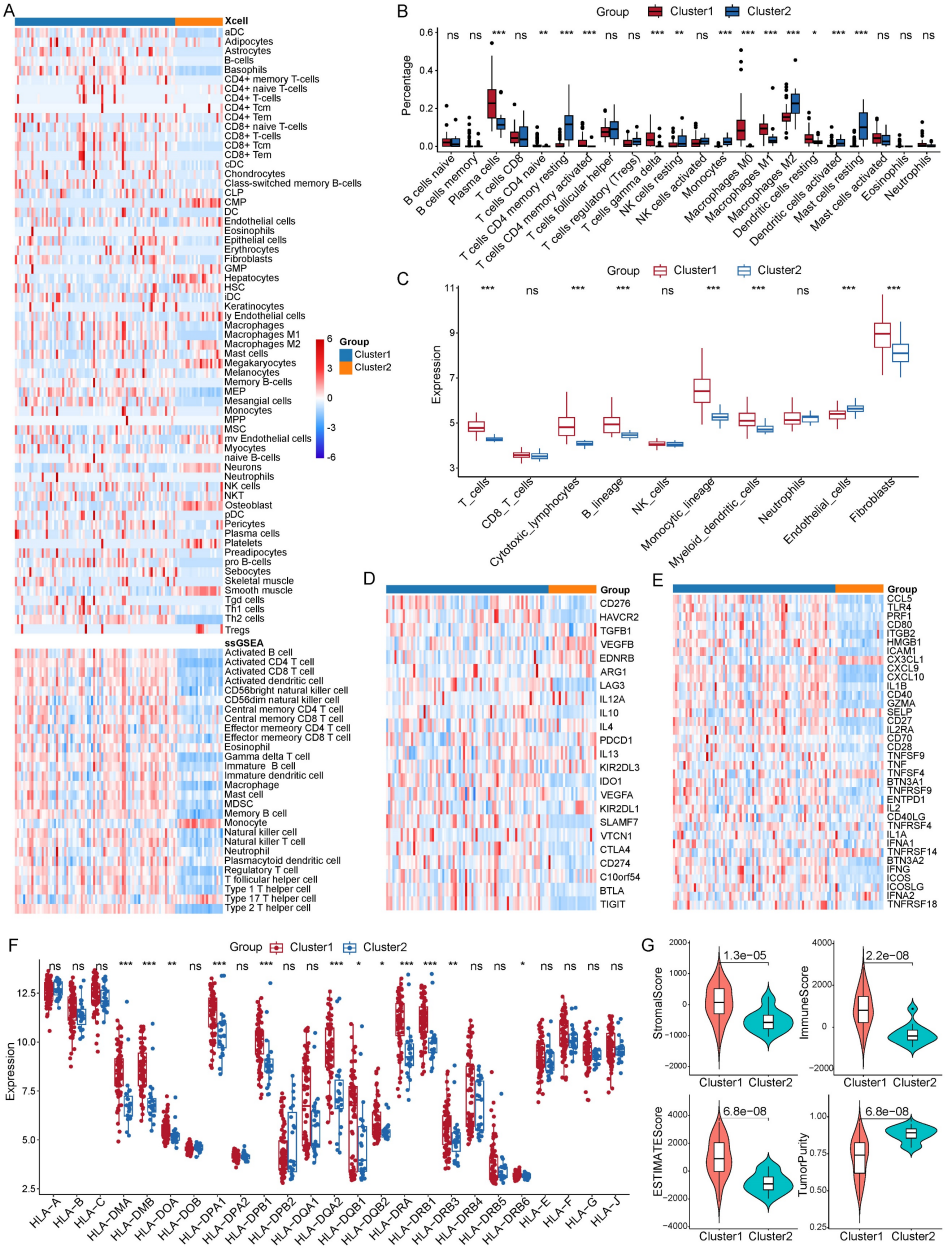
Comparison of immune microenvironments between two clusters. A Estimation of 64 and 28 immune cell infiltration using the xCell and ssGSEA method respectively. B Abundance of 22 immune cell types between the two clusters through CIBERSORT analysis. C Measurement of the abundance of immune cells using the MCP-counter algorithm. D Expression patterns of immune suppressive checkpoints across two clusters. E Expression patterns of immune active checkpoints across two clusters. F Expression profile of HLA family members across two clusters. G StromalScore, immune score, ESTIMATE score, and tumor purity between two clusters. *P<0.05, **P<0.01, ***P<0.001.

We sought to dissect the discrepancy in immune infiltration between the two clusters by calculating the abundance of infiltrating immune cells and molecules. In order to evaluate the abundance of these immune cells, we utilized four algorithms, specifically, Xcell, ssGSEA, CIBERSORT, and MCP-counter. Our findings, drawn from Xcell, indicated an enhanced infiltration level of various dendritic cell types in cluster 1 (Figure 5A). In the tumor immune microenvironment of patients within cluster 1, there were also increased infiltrating levels of B cells, fibroblasts, M1 macrophages, and Th2 cells. The ssGSEA algorithm pointed towards a higher abundance of B cells, T cells, and dendritic cells (Figure 5A). The enrichment of B cells, T cells, and macrophages was corroborated by the outputs of both CIBERSORT and MCP-counter methods (Figure 5B and 5C). Strikingly, a difference was observed in the expression levels of immune checkpoints between the two subgroups.

The expression level of ten immune suppressive checkpoints was also higher in cluster1 compared with cluster 2, including CD276, LAG3, IL10, PDCD1, CTLA4, CD274, and TIGIT (Figure 5D). Similarly, out of 36 active immune checkpoints, most of them highly expressed in cluster 1 such as CCL5, CD80, CD40, CXCL10, and GZMA (Figure 5E). Extensively exploring the expression patterns of the class I human leukocyte antigen (HLA) family members showed that the expression of 12 HLA family members was significantly upregulated in cluster 1 (Figure 5F). Moreover, we unraveled features of the immune microenvironment by calculating the ESTIMATE score and the immune score of each PNECs sample. Our analysis showed that the stromal score, immune score, and ESTIMATE score were significantly elevated in cluster 1, whereas the tumor purity was markedly decreased in cluster 1 (Figure 5G).

### Functional enrichment analysis of the DEGs in LNECs sub-types

**Figure 6:**
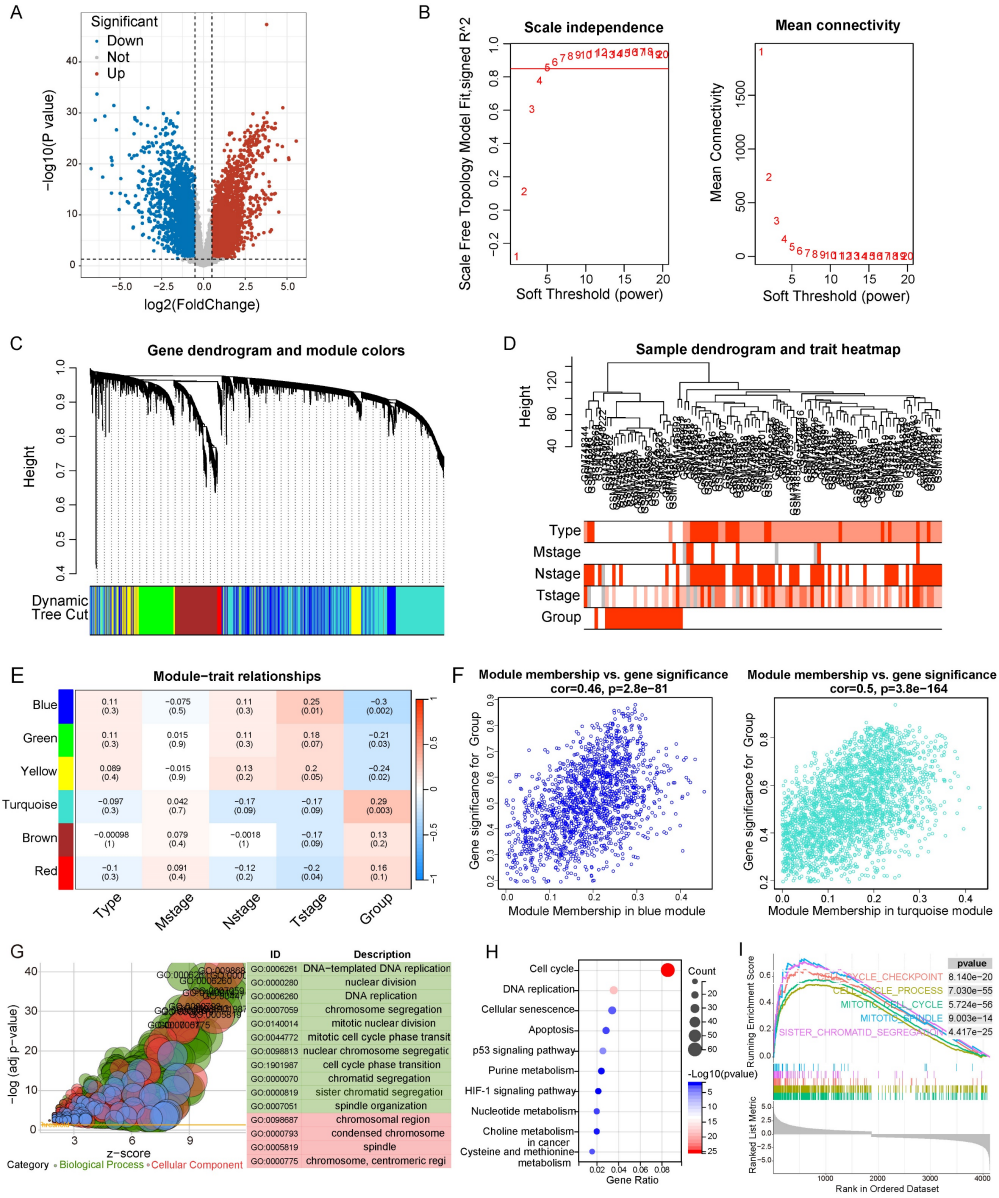
Differential gene analysis, WGCNA, and enrichment analysis of two clusters. A Volcano plot of 6332 DEGs in two clusters. B The scale-free fit index and the average connectivity of soft threshold power. C Hierarchical clustering gene tree based on weighted correlation coefficients. D Sample hierarchical clustering tree and clinical features. E Scatter plots of the blue and turquoise module. F GO analysis was performed using upregulated genes in blue and turquoise module. G KEGG analysis was performed using upregulated genes in blue and turquoise module. H GSEA analysis based on the genes in blue and turquoise module.

Initially, we analyzed the differentially expressed genes across the two clusters, yielding 6332 genes for subsequent examination (|logFC|>0.5 & p<0.05) (Figure 6A). We then employed WGCNA to build a gene co-expression network utilizing the DEGs (β = 5) (Figure 6B). Afterwards, a gene hierarchical clustering tree was derived through the dynamic hybrid cutting method, concurrently obtaining a sample hierarchical clustering tree with no pronounced outliers (Figure 6C and 6D). Upon completion, we discovered six gene modules, pinpointing the blue (Cor = 0.46, p = 2.8e-81) and turquoise (Cor = 0.50, p = 3.8e-164) modules as potential hub modules (Figure 6E and 6F). The upregulated genes identified within these two modules were selected for Gene Ontology (GO) and Kyoto Encyclopedia of Genes and Genomes (KEGG) analyses (Figure 6G and 6H). These genes were found enriched in multiple pathways pertinent to the cell cycle through both methods, including DNA replication, nuclear division, cell cycle, and p53 signaling pathway. Additionally, we implemented GSEA to perform an enrichment analysis, grounded on all genes found within the blue and turquoise modules, revealing an enrichment of cell cycle-related pathways such as cell cycle checkpoint, cell cycle process, and mitotic cell cycle (Figure 6I).

### Drug sensitivity analysis in LNECs sub-types

**Figure 7:**
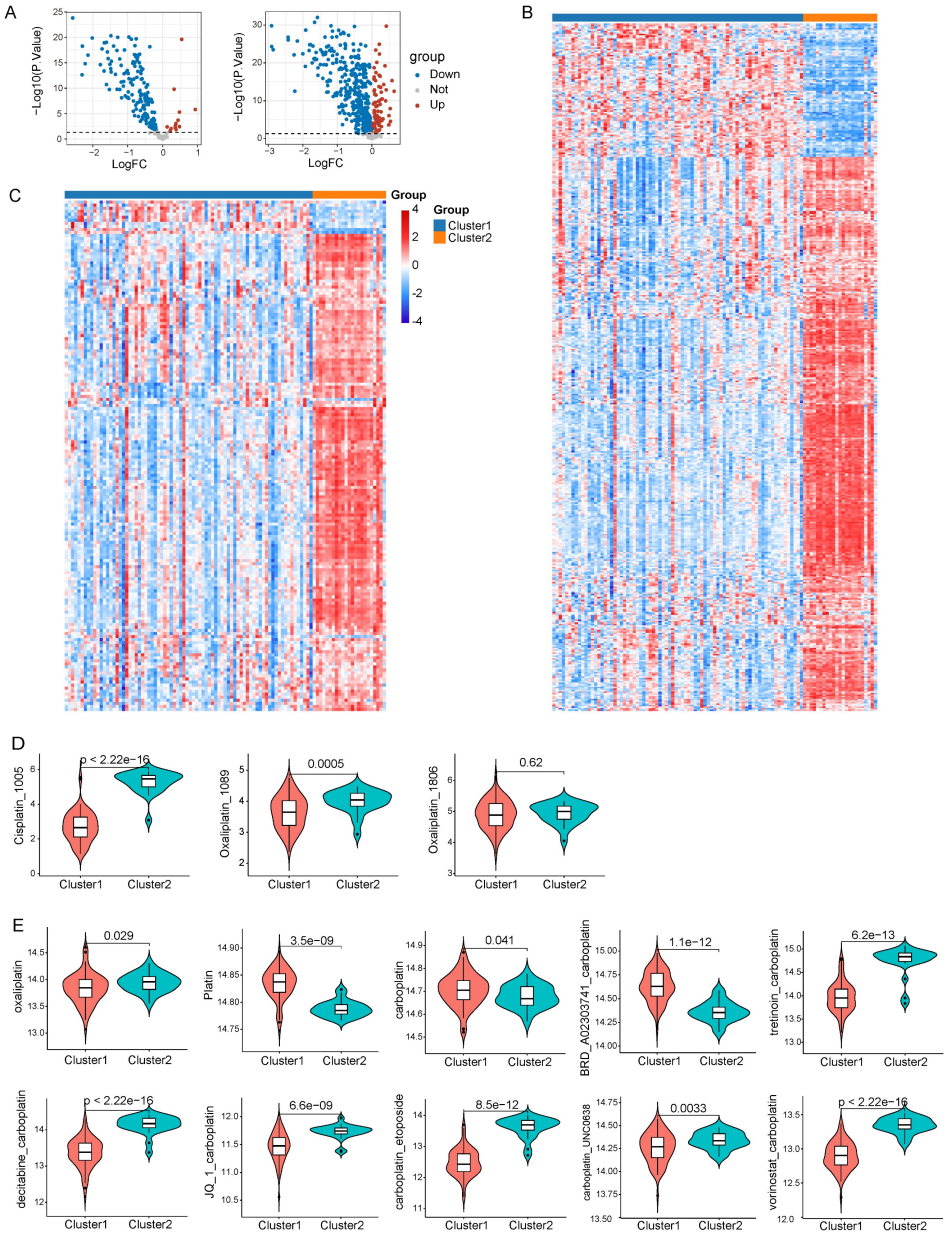
Drug sensitivity analysis based on GDSC and CTRP database. A Volcano plots of 168 and 458 differential drugs across two clusters based on GDSC and CTRP database respectively. B Heatmap reflected the IC50 score of 168 differential drugs based on GDSC database. C Heatmap reflected the IC50 score of 458 differential drugs based on CTRP database. D Drug sensitivity to three platinum-based drugs in GDSC database. E Drug sensitivity to ten platinum-based chemotherapy strategies in CTRP database.

Given the pivotal role that chemotherapy plays in the treatment of LNECs, we utilized the R package “oncoPredict” to forecast clinical responses of each sample to chemotherapy. We drew expression data of cell lines and IC50 data for drugs from GDSC and CTRP databases. By employing the “oncoPredict” algorithm, we generated a drug sensitivity score per sample based on the acquired data from GDSC and CTRP. Subsequently, the differential drug sensitivity score between the two clusters was analyzed by “limma” algorithm, with a p<0.05 set as the threshold for determining effective drugs (Figure 7A). Relative to cluster 2, our results indicated that cluster 1 showcased sensitivity to 155 drugs and resistance to 13 drugs, as per the GDSC database (Figure 7B). The CTRP database suggested that 369 drugs were presumably more effective on cluster 1, while 89 drugs showed heightened sensitivity specifically to cluster 2 (Figure 7C). Given the significance of platinum in chemotherapy for tumor treatment, we scrutinized clinical responses of each sample to platinum-based chemotherapy. We chose three platinum-based drugs from GDSC database and our results divulged that patients in cluster 1 presented sensitivity to both Cisplatin_1005 and Oxaliplatin_1089 (Figure 7D). Out of the ten platinum-based chemotherapy strategies offered by the CTRP database, cluster 1 patients showed sensitivity towards oxaliplatin while they displayed resistance to platin treatment (Figure 7E). Noteworthy is that despite the apparent inefficacy of carboplatin or the combination of carboplatin and BRD_A02303741 on cluster 1 patients, other carboplatin-based chemotherapy strategies exhibited promising responses (Figure 7E).

### ALDH1L2 knockdown represses the malignant behavior of PNECs *in vitro*

**Figure 8:**
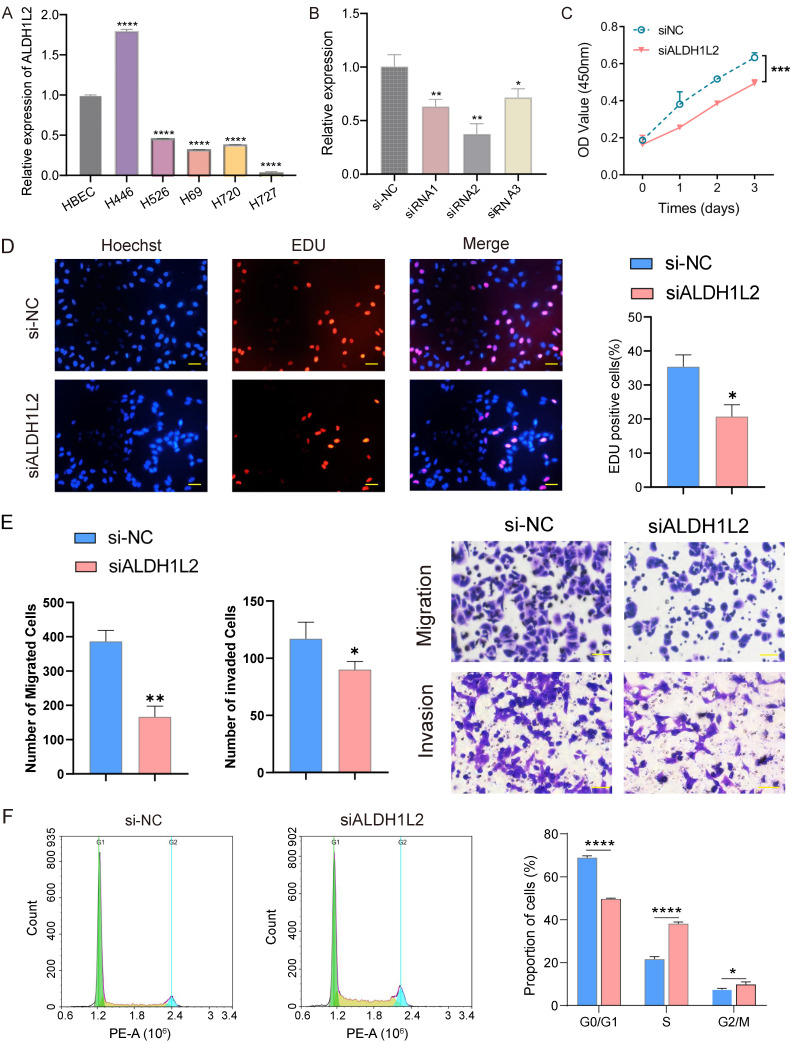
The expression level and biological functions of ALDH1L2 in PNECs cell lines. A The mRNA level of ALDH1L2 in multiple PNECs cell lines and immortalized lung epithelial cell. B The expression of ALDH1L2 in the NC and three ALDH1L2 knockdown groups of NCI-H446 cells. C The effect of ALDH1L2 knockdown on NCI-H446 cells via CCK-8 analysis. D EdU assay showed the effect of ALDH1L2 knockdown on the proliferation of NCI-H446 cells. E The migration and invasion ability of the NC and ALDH1L2 deficient NCI-H446 cells. F The effect of ALDH1L2 knockdown on the cell cycle in NCI-H446 cells. *P<0.05, **P<0.01, ***P<0.001, ****P<0.0001.

We began our investigation by measuring the relative expression level of the three core genes *in vitro* using qRT-PCR. Compared with the HBEC cell line, ALDH1L2 was notably overexpressed in NCI-H446 cells (SCLC cell line) but exhibited a lower expression level in NCI-H720 and NCI-H727 cells (two PCs cell lines) (Figure 8A). In contrast, MTHFD2 and SHMT2 displayed diminished expression levels in both SCLC and PCs cell lines (Supplement Figure 2). Noteworthily, MTHFD2 and SHMT2 generally had higher expression levels in SCLC cell lines as opposed to PCs cell lines (Supplement Figure 2). Consequently, we investigated the biological function of ALDH1L2 in the NCI-H446 cell line, employing siRNA to suppress the expression of ALDH1L2 in NCI-H446 cells (Figure 8B). Our results demonstrated ALDH1L2's role in PNECs proliferation, revealing that the suppression of ALDH1L2 could significantly inhibit the ability of the cell to proliferate (Figure 8C). It was also found that diminishing ALDH1L2 expression significantly hindered the level of DNA replication *in vitro* (Figure 8D). We examined the impact of ALDH1L2 deficiency on migration and invasion *in vitro*. Then, we examined the impact of ALDH1L2 deficiency on migration and invasion *in vitro*. The results indicated a significant impairment in both migration and invasion abilities following the inhibition of ALDH1L2 (Figure 8E). Further, an analysis of the cell cycle highlighted that, in comparison to the control group, ALDH1L2 knockdown notably increased the proportion of cells in the S phase (Figure 8F).

## Discussion

Folate metabolism is one of the most crucial part of the metabolic alterations in tumor cells, often resulting in the poor prognosis of multiple malignant tumors^8^, ^19^, ^29^, ^30^. Previous studies have confirmed the pivotal role of targeting folate metabolism in cancer treatment^31^-^34^. Therefore, efforts to investigate the prognostic value and biological function of folate metabolism for PNECs are still of paramount importance. In present study, we evaluated the expression pattern of folate metabolism related genes in PNECs using both bulk RNA-seq and scRNA-seq data. Subsequently, we explored the prognostic role of these genes and identified core genes using through four machine learning methods. A molecular classification was constructed which segments the patients into two clusters. We then elucidated differences in immune infiltration characteristics, potential biological mechanisms, and drug sensitivity between the two sub-types. Finally, ALDH1L2 was selected and its biological role in promoting tumor progression was validated *in vitro*.

A previous study has confirmed the elevated expression level of five folate metabolism enzymes in SCLC, including *MTHFD2, PGDH3, SHMT2, MTHFD1* and* TYMS*^35^. Nevertheless, the expression patterns of other folate metabolism enzymes in SCLC and their levels of expression in other PNECs types remain undetermined. Moreover, evidence indicating the expression patterns of these enzymes at a single-cell level continues to be inadequate. Our study found that 20 genes exhibited differential expression in PNEC tumor tissues, including 18 upregulated and 2 downregulated genes. Most of these genes were found expressed in epithelial cells, while certain enzymes demonstrated high expression levels in immune cells, such as *MTHFR* in monocytes, *CHDH* in macrophages, and *GNMT* in B cells. The result based on bulk RNA-seq showed that the expression pattern of folate metabolism related genes in PCs significantly diverged from that in LCNEC and SCLC, but closely resembled the normal tissue (Supplement Figure 1C). This similarity between PCs and normal lung was also observed in epithelial cells derived from scRNA-seq data.

The prognostic value of folate metabolism enzymes in lung adenocarcinoma had been proved in our previous study^28^. However, another study argued that five folate metabolism enzymes exhibited no correlation with OS in lung squamous cell carcinoma and SCLC^35^. Our findings indicated that all folate metabolism genes were connected to the prognosis of PNECs, and we identified 16 as prognostic genes. This difference perhaps stemmed from the larger number of samples (n = 101 vs n = 37) and the testing method (RNA-seq vs IHC). From 16 prognostic genes, we isolated three core genes (*MTHFD2, SHMT2,* and* ALDH1L2*) using four methods and constructed a molecular classification. Cluster 1 was characterized by a shorter survival time, advanced T stage and a predominance of SCLC and LCNEC samples. These features were consistent with the clinical evidence suggesting that compared to PCs, SCLC and LCNEC proved more malignant and exhibited lower survival rates due to rapid tumor cell proliferation.

The efficacy of immunotherapy in SCLC has been confirmed in several clinical trails^36^, ^37^. Unfortunately, there is a lack of substantial evidence to affirm the clinical benefits of immunotherapy in LCNEC and PCs. T cells and macrophages have been validated playing an important role in tumor progression and treatment^23^, ^25^, ^26^. In our study, the results showed a considerably higher abundance of T cells and macrophages in cluster 1 compared to cluster 2. The expression level of specific immune checkpoints and HLA family members was increased in cluster1. Previous studies revealed that the expression level of PD-L1 in SCLC approximated to LCNEC and exceeded those of PCs^38^-^40^. In addition, the immune score, stromal score, and ESTIMATE score were higher in Cluster 1 compared to Cluster 2. These results implied that the immune infiltration patterns between SCLC and LCNEC are similar, creating a distinct contrast with PCs (Supplement Figure 3). From these findings, the efficacy of immunotherapy in LCNEC can be reasonably deduced.

Among the PNECs sub-types, PCs was characterized by a low proliferation rate of tumor cells and a favorable prognosis. Our research also observed advanced T-stage and poor survival in Cluster 1, where the samples consisted predominantly of SCLC and LCENC. Aberration in cell cycle causes the uncontrolled cell proliferation and eventually lead to tumor formation, which makes inhibiting tumor cell cycle the fundamentally principle in cancer treatment^41^, ^42^. Platinum-based chemotherapy is remains one of the most crucial therapeutic strategies of PNECs, especially in treating SCLC. Platinum-based drugs, a subset of cell cyclin-specific drugs, exerts their anti-tumor function by forming Pt-DNA adducts. Our study reveals that cell cycle related pathways were upregulated in Cluster 1, suggesting it may be more responsive to platinum-based chemotherapy. This inference was further substantiated by the drug sensitivity analysis and consistent to the clinical evidence that platinum-based drugs are comparatively more effective in treating SCLC and LCNEC than PCs^43^.

Aldehyde dehydrogenase 1 family member 2 (ALDH1L2), a 10-formyltetrahydrofolate (10-fTHF) dehydrogenase, can catalyzes the 10-fTHF dehydrogenase reactions that produce mitochondrial NADPH, thereby exerting antioxidant functions^44^-^46^. The expression of ALDH1L2 have been observed increased in multiple tumor types^47^. Prior research demonstrated that ALDH1L2 is highly expressed in colorectal cancer and is correlated with the poor prognosis^48^, ^49^. The pro-tumorigenic functions of ALDH1L2 was also observed in in pancreatic cancer^50^. Contrarily, a recent study found that enhancement of ALDH1L2 expression could inhibit the metastatic capability of breast cancer cells^44^. Our study indicated high expression of ALDH1L2 in SCLC cell lines, with the expression generally exceeding that in PCs cell lines. Subsequent results suggested that compared with the control group, ALDH1L2 knockdown markedly repressed the proliferation and migration capacity of tumor cells. Flow cytometry revealed that ALDH1L2 deficiency increased cell proportion in S phase while diminishing that in G0/G1 phase, indicating that ALDH1l2 deficient tumor cells were arrested at S phase.

The present study was affected by several significant limitations. First, only one bulk RNA-seq data was obtained due to the rarity of PNECs, resulting in a deficit of external validation datasets. Second, the scRNA-seq data of SCLC derived from xenograft tumor models causing a loss of TME information. Third, the expression level of ALDH1L2 was not evaluated in tissues because of the limited PNECs samples. Additional large-scale datasets were needed to verify the role of folate metabolism related genes inPNECs, and biological mechanisms of ALDH1L2 needed to be further explored by experiments *in vivo* and *in vitro*.

In conclusion, we constructed a new gene signature centered on folate metabolism enzymes. Our research revealed differential expression of folate metabolism related genes between PNECs tumor tissues and normal lung tissues, exhibiting prognostic relevance. PNECs samples could be divided into two clusters which displayed the significant disparities in prognosis, immune infltration, biological mechanisms, and drug sensitivity. For the first time, the pro-tumorigenic functions of ALDH1L2 in PNECs were confirmed *in vitro* experiments. Our study not only enriched the understanding of PNECs pathogenesis, but also provide a new insight for therapeutic strategies for PNECs patients.

## Supplementary Material

Supplementary figures.

## Figures and Tables

**Table 1:** Clinicopathological Characteristics of PNECs.

	Cluster 1 (n = 78)	Cluster 2 (n = 23)	P
Gender			0.001
Male	71(91.0)	13(56.5)	
Female	7(9.0)	10(43.5)	
T stage			0.001
T1	14(17.9)	13(56.5)	
T2	30(38.5)	9(39.1)	
T3	15(19.2)	0(0)	
T4	13(17.7)	1(4.3)	
TX	6(7.7)	0(0)	
Lymph nodes			0.852
N0	36(46.2)	9(39.1)	
N1	14(17.9)	5(21.7)	
N2	18(23.1)	7(30.4)	
N3	8(10.3)	2(8.7)	
NX	2(2.6)	0(0)	
Metastasis			0.529
M0	69(88.5)	22(95.7)	
M1	6(7.7)	1(4.3)	
MX	3(3.8)	0(0)	
Type			
PC	2(2.6)	22(95.7)	<0.001
LCNEC	55(70.5)	1(4.3)	
SCLC	21(26.9)	0(0)	
