# Supplementary material for: Constructing a folate metabolism gene signature for predicting prognosis in pulmonary neuroendocrine carcinomas
Source: J Cancer. 2024 Oct 14;15(19):6256–72. doi: 10.7150/jca.102186 (PMC11540503; doi:10.7150/jca.102186)
Supplement: Supplementary file 1 — Supplementary figures. [file jcav15p6256s1.pdf]

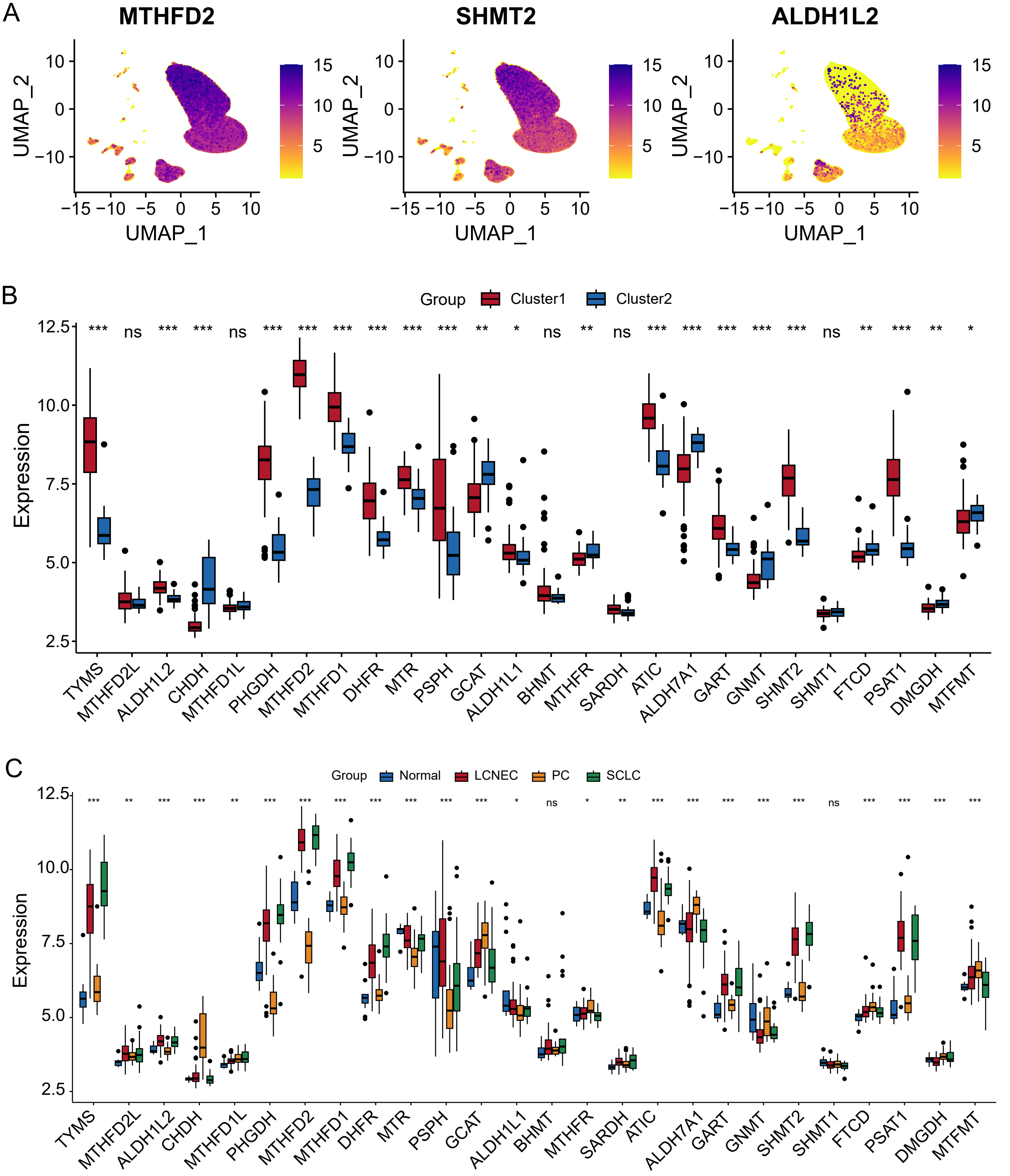
A
MTHFD2
SHMT2
ALDH1L2
UMAP_1
UMAP_2
B
Group Cluster1 Cluster2
Expression
TYMS MTHFD2L ALDH1L2 CHDH MTHFD1L PHGDH MTHFD2 MTHFD1 DHFR MTR PSPH GCAT ALDH1L1 BHMT MTHFR SARDH ATIC ALDH7A1 GART GNMT SHMT2 SHMT1 FTCD PSAT1 DMGDH MTFMT
C
Group Normal LCNEC PC SCLC
Expression

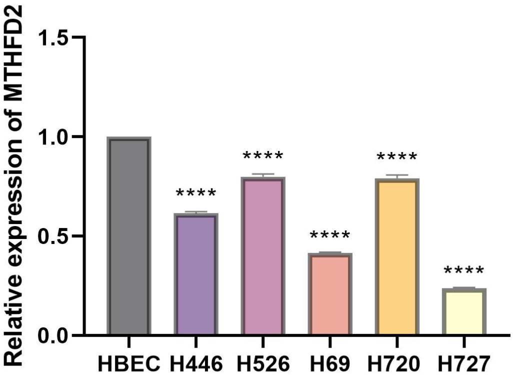

Relative expression of MTHFD2
1.5
1.0
0.5
0.0
****
****
****
****
****
HBEC
H446
H526
H69
H720
H727

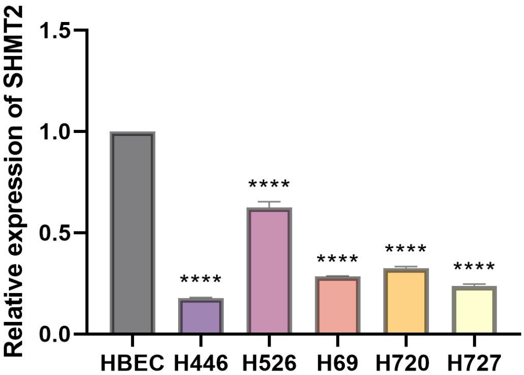

Relative expression of SHMT2
1.5
1.0
0.5
0.0
****
****
****
****
****
HBEC
H446
H526
H69
H720
H727

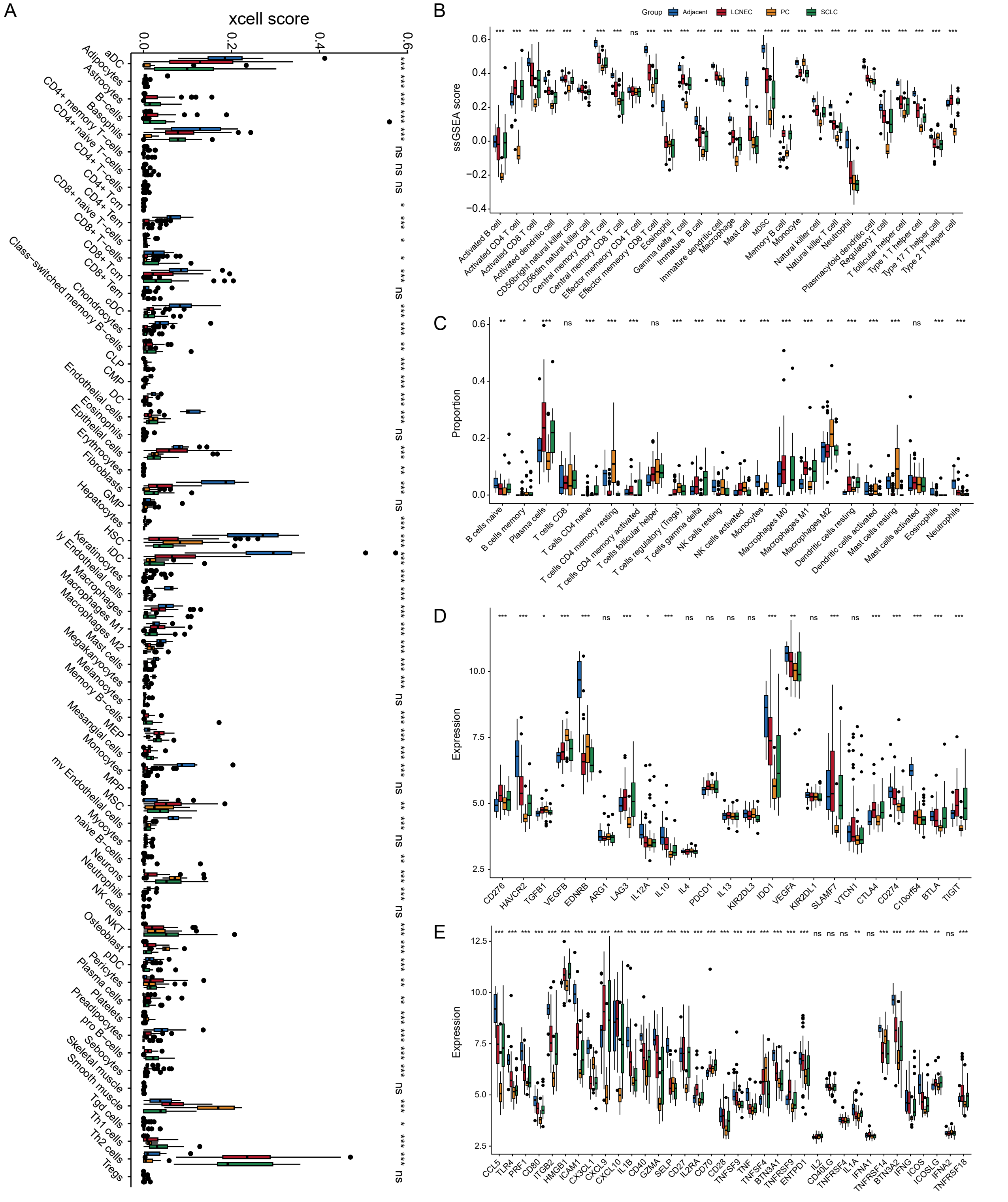

A
xcell score
0.0
0.2
0.4
0.6
aDC
Adipocytes
Astrocytes
B−cells
Basophils
CD4+ memory T−cells
CD4+ naive T−cells
CD4+ T−cells
CD4+ Tcm
CD4+ Tem
CD8+ naive T−cells
CD8+ T−cells
CD8+ Tcm
CD8+ Tem
cDC
Chondrocytes
Class−switched memory B−cells
CLP
CMP
DC
Endothelial cells
Eosinophils
Epithelial cells
Erythrocytes
Fibroblasts
GMP
Hepatocytes
HSC
iDC
Keratinocytes
ly Endothelial cells
Macrophages
Macrophages M1
Macrophages M2
Mast cells
Megakaryocytes
Melanocytes
Memory B−cells
MEP
Mesangial cells
Monocytes
MPP
MSC
mv Endothelial cells
Myocytes
naive B−cells
Neurons
Neutrophils
NK cells
NKT
Osteoblast
pDC
Pericytes
Plasma cells
Platelets
Preadipocytes
pro B−cells
Sebocytes
Skeletal muscle
Smooth muscle
Tgd cells
Th1 cells
Th2 cells
Tregs
B
Group
Adjacent
LCNEC
PC
SCLC
ssGSEA score
0.6
0.4
0.2
0.0
−0.2
−0.4
Activated B cell
Activated CD4 T cell
Activated CD8 T cell
Activated dendritic cell
CD56bright natural killer cell
CD56dim natural killer cell
Central memory CD4 T cell
Central memory CD8 T cell
Effector memeory CD4 T cell
Effector memeory CD8 T cell
Eosinophil
Gamma delta T cell
Immature B cell
Immature dendritic cell
Macrophage
Mast cell
MDSC
Memory B cell
Monocyte
Natural killer cell
Natural killer T cell
Neutrophil
Plasmacytoid dendritic cell
Regulatory T cell
T follicular helper cell
Type 1 T helper cell
Type 17 T helper cell
Type 2 T helper cell
C
Proportion
0.6
0.4
0.2
0.0
B cells naive
B cells memory
Plasma cells
T cells CD8
T cells CD4 naive
T cells CD4 memory resting
T cells CD4 memory activated
T cells follicular helper
T cells regulatory (Tregs)
T cells gamma delta
NK cells resting
NK cells activated
Monocytes
Macrophages M0
Macrophages M1
Macrophages M2
Dendritic cells resting
Dendritic cells activated
Mast cells resting
Mast cells activated
Eosinophils
Neutrophils
D
Expression
10.0
7.5
5.0
2.5
CD276
HAVCR2
TGFB1
VEGFB
EDNRB
ARG1
LAG3
IL12A
IL10
IL4
PDCD1
IL13
KIR2DL3
IDO1
VEGFA
KIR2DL1
SLAMF7
VTCN1
CTLA4
CD274
C10orf54
BTLA
TIGIT
E
Expression
12.5
10.0
7.5
5.0
2.5
CCL5
TLR4
PRF1
CD80
ITGB2
HMGB1
ICAM1
CX3CL1
CXCL9
CXCL10
IL1B
CD40
GZMA
SELP
CD27
IL2RA
CD70
CD28
TNFSF9
TNF
TNFSF4
BTN3A1
TNFRSF9
ENTPD1
IL2
CD40LG
TNFRSF4
IL1A
IFNA1
TNFRSF14
BTN3A2
IFNG
ICOS
ICOSLG
IFNA2
TNFRSF18

Supplemental Figure 1. A Feature plots of three core genes. B Expression patterns of folate metabolism related genes across two clusters. C Expression patterns of folate metabolism related genes across tumor types.

Supplemental Figure 2. The mRNA level of MTHFD2 and SHMT2 in multiple PNECs cell lines and immortalized lung epithelial cell.

Supplemental Figure 3. Immune microenvironments features across tumor types. Estimation of immune cell infiltration using the xCell (A), ssGSEA (B), and CIBERSORT (C) method respectively. Expression patterns of immune suppressive (D) and active (E) checkpoints across tumor types.
